# Blood Lead Level Is Negatively Associated With Bone Mineral Density in U.S. Children and Adolescents Aged 8-19 Years

**DOI:** 10.3389/fendo.2022.928752

**Published:** 2022-07-01

**Authors:** Aiyong Cui, Peilun Xiao, Baoliang Hu, Yuzhuo Ma, Zhiqiang Fan, Hu Wang, Fengjin Zhou, Yan Zhuang

**Affiliations:** ^1^Department of Pelvic and Acetabular Surgery, HongHui Hospital, Xi’an Jiaotong University, Xi’an, China; ^2^Department of Orthopaedics, The Fifth Affiliated Hospital of Sun Yat-Sen University, Zhuhai, China; ^3^Department of gastroenterology surgery, HongHui Hospital, Xi’an Jiaotong University, Xi’an, China; ^4^Department of Orthopedics, HongHui Hospital, Xi’an Jiaotong University, Xi’an, China

**Keywords:** lead, bone mineral density, children, adolescent, NHANES

## Abstract

**Context:**

The relationship of lead (Pb) exposure with bone health in children and adolescents remains controversial.

**Objection:**

We aimed to investigate the association of blood lead levels (BLL) with bone mineral density (BMD) in American children and adolescents using data from the National Health and Nutrition Examination Survey (NHANES), 2005-2010.

**Methods:**

We analyzed 5,583 subjects aged 8-19 years (mean age, 13.49 ± 3.35 years) from the NHANES 2005-2010. BLL was tested using inductively coupled plasma mass spectrometry. BMD was measured by dual-energy X-ray absorptiometry (DXA) at the lumbar spine, total femur, and femur neck. Multivariate linear regression models were used to explore the association between BLL and BMD, adjusting for age, gender, race/ethnicity, poverty income ratio (PIR), body mass index (BMI), serum calcium, and serum phosphorus.

**Results:**

BLL was negatively correlated with BMD at different sites of interest in children and adolescents. For every 1mg/dl increase in BLL, the BMD of the total spine, total hip, and femoral neck decreased by 0.011 g/cm^2^, 0.008 g/cm^2^, and 0.006 g/cm^2^. In addition, Pb affected the lumbar spine more than the femur. The effect estimates were stronger in girls than boys at the lumbar spine (*P* for interaction= 0.006). This negative association remained significant in American children and adolescents after excluding individuals with BLL more than 3.5 ug/dl.

**Conclusion:**

Our study indicates that BLL is negatively correlated with BMD at different sites of interest in children and adolescents aged 8-19 years, even in the reference range. More research is needed to elucidate the relationships between Pb and bone health in children and adolescents, including specific mechanisms and confounding factors like race/ethnicity, gender, and age.

## Introduction

Osteoporosis is a multifactorial skeletal disease characterized by low bone mineral density (BMD) and increased fragility fracture risks. In 2010, more than 24 million people aged 50 and older in the United States were estimated to have osteoporosis (1). The peak bone mass (PBM) in adolescence and bone loss with age are two major factors in the process of osteoporosis. PBM in adolescence has been shown to be a significant predictor of osteoporosis in old age (2). Population-based studies have proved that a 10% increase in PBM could reduce fracture risk by 50% in old age (3). Several factors influencing PBM formation have been explored, including race, gender, genetics, and lifestyle (4, 5).

Lead (Pb), listed as one of the ten most harmful metals by the World Health Organization (WHO), has been reported to be associated with osteoporosis (6–10). Despite a significant decline in blood lead (BLL) in the U.S. population, many sources of Pb exposure still exist including drinking water and contaminated soil particles (11, 12). Studies have explored the molecular mechanism of Pb damage to bone health. Bone is the main site of lead storage in the body (13). Some studies suggest that calcium in hydroxyapatite crystals can be exchanged by Pb, which leads to a decrease in bone mass (14). Moreover, Pb has a substantial regulatory effect on growth plate chondrocytes and inhibits the process of endochondral bone formation (14). Previous studies have found the adverse effects of Pb on bone health in adults (10, 15, 16). However, evidence for the association between Pb exposure and BMD in children and adolescents was scarce and inconsistent. An early study in America stated that children with higher BLL were associated with an increased BMD. However, it recruited smaller samples, which may result in an unreliable conclusion (17). A recent study conducted by Li et al. (18) showed an N-shaped curve association between BLL and BMD in children and adolescents.

Pb exposure is especially hazardous for children since they are more likely to absorb Pb than adults (19). In order to prevent future osteoporosis, it is crucial to explore the association of Pb exposure with BMD in children and adolescents, as they acquire most of their PBM at the end of puberty (20). Therefore, the focus of our study is to evaluate the correlation of BLL with BMD in American children and adolescents. We hypothesize that BLL is negatively associated with BMD in children and adolescents.

## Materials and Methods

### Study Population

National Health and Nutrition Examination Survey (NHANES), using a stratified, multi-stage random sampling design, is a nationally representative nutrition survey of general populations in the United States. Three consecutive cycles of NHANES (2005–2006, 2007–2008, 2009–2010) were selected since the femur BMD of American children and adolescents are only available in these cycles. We initially included 7,313 subjects aged 8–19 years from NHANES 2005-2010. After excluding 1,729 subjects with missing BMD (n = 1,157) and blood lead (n = 573) data, this study includes 5,583 eligible subjects for analysis.

### Variables

The venous blood of the subjects was collected during the interview. Whole blood specimens were processed and shipped to the National Center for Environmental Health for testing and analysis. BLL was tested using inductively coupled plasma mass spectrometry. A detailed manual of laboratory procedures is available on the NHANES website (21). The dependent variables were the total femur, femur neck, and total spine BMD measured by DXA (Hologic, Inc., Bedford, Massachusetts). Total spine BMD was the average BMD of the L1 to L4. The left hip was routinely scanned to estimate the total femur, and femoral neck BMD. The right hip was selected for scanning if participants had left hip arthroplasty or metal object implantation. BMD values were collected and standardized by professionals.

Based on previous studies (18, 22–24), confounders that may influence BMD were chosen to eliminate potential effects on the results. We also performed a multicollinearity analysis of these covariates and did not find the presence of multicollinearity. Finally, the following covariates were collected and adjusted, including gender, age, race/ethnicity, PIR (poverty income ratio), body mass index (BMI), serum calcium, and serum phosphorus. Details on the covariates can be found on the NHANES website (http://www.cdc.gov/nchs/nhanes/).

### Statistical Analysis

All statistical analyses were performed using Package R and EmpowerStats (http://www.empowerstats.com), with a complex weighted sampling design from NHANES. The characteristics of participants were described according to quartile of BLL (Categories 1: 0.18-0.59 ug/dl; Categories 2: 0.59-0.82 ug/dl; Categories 3: 0.82-1.20 ug/dl; Categories 4: >1.20 ug/dl). We used percentages for categorical variables and means ± standard deviations for continuous variables. To compare the difference among the groups, we employed the weighted χ2 test and linear regression model to analyze categorical and continuous variables, respectively. Weighted multivariate linear regression models were used to assess the association between BLL and the total femur, femur neck, and total spine BMD. An unadjusted model was created first (Model 1), and then a minimally adjusted model (Model 2) was built after adjusting age, gender, and race/ethnicity. Finally, a fully adjusted model (Model 3) was calculated, adjusting for age, gender, race/ethnicity, PIR, BMI, serum calcium, and serum phosphorus. Then stratified analyses were performed by age, gender, and race/ethnicity, and their interactions were tested. As the current normal reference value range of BLL in the U.S. is 3.5 ug/dl (25), we performed another weighted multivariate linear regression analysis after excluding individuals whose BLL was more than 3.5 ug/dl to exclude the influence of the children and adolescents with very high BLL. *P* values less than 0.05 were considered statistically significant in the analyses.

## Results

### Study Participants and Baseline Characteristics

A total of 5,583 participants with a mean age of 13.49 ± 3.35 years were enrolled. BLL values were detected in all participants, with a mean of 1.04 ± 0.87 ug/dL. In this study, 59.28% of the participants were non-Hispanic white, 13.94% were non-Hispanic black, 13.77% were Mexican American, and 7.16% were other races (including multiracial population), and 5.85% were other Hispanic. The weighted characteristics of participants were described according to quartile of BLL (Categories 1: 0.18-0.59 ug/dl; Categories 2: 0.59-0.82 ug/dl; Categories 3: 0.82-1.20 ug/dl; Categories 4: >1.2 ug/dl), as listed in **Table 1**. According to the BLL quartiles, the participants’ characteristics were significantly different except for cholesterol and total protein. Participants in the highest quartile of BLL were more likely to be younger, men, and non-Hispanic Blacks. They had a higher value of serum uric acid and a lower value of PIR, BMI, total femur, femur neck, and total spine BMD.

**Table 1:** Characteristics of the study population based on BLL quartiles.

		Blood lead (μg/dL)				
	total	Q1 (0.18-0.59)	Q2 (0.59-0.82)	Q3 (0.82-1.20)	Q4 (>1.20)	*P* value
Number of subjects (n)	5583	1386	1386	1391	1420	
Age (years)	13.49 ± 3.35	14.09 ± 3.06	13.65 ± 3.36	13.28 ± 3.45	12.70 ± 3.43	<0.001
Gender (%)						<0.001
Men	53.27%	36.74%	53.72%	60.84%	66.86%	
Women	46.73%	63.26%	46.28%	39.16%	33.14%	
Race/ethnicity (%)						<0.001
Mexican American	13.77%	14.41%	11.98%	12.77%	16.39%	
Other Hispanic	5.85%	5.11%	5.36%	6.20%	7.11%	
Non-Hispanic White	59.28%	67.64%	62.35%	56.40%	47.00%	
Non-Hispanic Black	13.94%	8.39%	12.89%	15.81%	20.87%	
Other Race (Including Multi-Racial)	7.16%	4.45%	7.42%	8.83%	8.63%	
PIR	2.61 ± 1.60	3.00 ± 1.60	2.82 ± 1.55	2.41 ± 1.58	2.01 ± 1.49	<0.001
Blood urea nitrogen (mmol/L)	3.67 ± 0.96	3.62 ± 0.90	3.64 ± 0.93	3.73 ± 1.09	3.71 ± 0.93	0.007
Serum total calcium (mmol/L)	2.41 ± 0.06	2.41 ± 0.07	2.41 ± 0.06	2.42 ± 0.07	2.42 ± 0.06	<0.001
Serum phosphorus (mmol/L)	1.42 ± 0.18	1.40 ± 0.17	1.42 ± 0.18	1.44 ± 0.19	1.44 ± 0.18	<0.001
Cholesterol (mmol/L)	4.24 ± 0.64	4.21 ± 0.69	4.24 ± 0.64	4.24 ± 0.61	4.27 ± 0.60	0.188
Total protein (g/L)	7.20 ± 0.33	7.19 ± 0.34	7.20 ± 0.35	7.19 ± 0.33	7.21 ± 0.30	0.508
Serum uric acid (mmol/L)	301.80 ± 58.97	291.10 ± 60.81	303.55 ± 59.76	304.96 ± 55.87	310.74 ± 56.69	<0.001
BMI (kg/m^2^)	22.49 ± 5.68	23.61 ± 5.91	22.95 ± 5.81	22.15 ± 5.53	21.27 ± 5.19	<0.001
Total femur BMD (g/cm^2^)	0.90 ± 0.19	0.92 ± 0.18	0.92 ± 0.19	0.89 ± 0.19	0.87 ± 0.19	<0.001
Femur neck (g/cm^2^)	0.84 ± 0.17	0.86 ± 0.16	0.85 ± 0.18	0.82 ± 0.17	0.81 ± 0.17	<0.001
Total spine BMD (g/cm^2^)	0.85 ± 0.20	0.90 ± 0.19	0.86 ± 0.20	0.83 ± 0.20	0.79 ± 0.20	<0.001
Blood lead (μg/dL)	1.04 ± 0.87	–	–	–	–	–

Mean ± SD for continuous variables: the P value was calculated by the weighted linear regression model. (%) for categorical variables. The P value was calculated by the weighted chi-square test. BLL Blood lead levels. BMD bone mineral density. PIR poverty income ratio. BMI body mass index.

### Correlation Between Blood Lead and Bone Mineral Density

#### Overall

**Figure 1:**
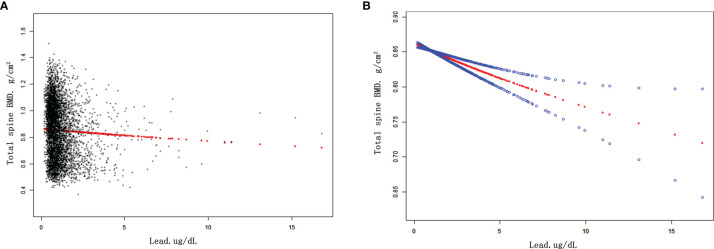
The associations between BLL and total spine BMD. **(A)** Each black point represents a sample. **(B)** Red line represents the smooth curve fit between variables. Blue lines represent the 95% of confidence interval from the fit. Age, gender, race/ethnicity, PIR, BMI, serum calcium, serum phosphorus were adjusted. Abbreviations: BLL, blood lead levels. PIR, poverty income ratio. BMI, body mass index. BMD, bone mineral density.

The results of weighted multivariate regression analyses for the association between BLL and BMD in American children and adolescents were shown in **Table 2**. BLL was negatively correlated to BMD in the three models at all sites of interest. After adjusting all covariates (model 3), BLL was negatively correlated to total spine BMD (β= -0.011 95% CI: -0.015, -0.007, *P*<0.001), total femur BMD (β= -0.008 95% CI: -0.012, -0.003, *P*=0.001), and femur neck (β= -0.006 95% CI: -0.010, -0.002, *P*=0.005). Smooth curve fittings of the association between BLL and BMD at lumbar spine and femur were shown in **Figure 1** and **Appendices 1**, **2**. We converted BLL from a continuous variable to a categorical variable (quartiles). Individuals in the highest BLL quartile had a lower mean BMD than those in the lowest quartile, with -0.018 g/cm^2^ lower BMD at total spine (β= -0.018 95% CI: -0.027, -0.010, *P*<0.001), -0.013 g/cm^2^ at the total femur (β= -0.013 95% CI: -0.022, -0.004, *P*<0.001), and -0.010 g/cm^2^ at femur neck (β= -0.010 95% CI: -0.018, -0.009, *P*=0.029) (**Table 2**). This association remained significant in American children and adolescents after excluding participants with BLL more than 3.5 ug/dl (**Table 3**).

**Table 2:** Results of weighted linear regression modeling for associations of the BLL with BMD at different sites.

	Total spine			Total femur			Femur neck		
	Model 1 β (95% CI) *P* value	Model 2 β (95% CI) *P* value	Model 3 β (95% CI) *P* value	Model 1 β (95% CI) *P* value	Model 2 β (95% CI) *P* value	Model 3 β (95% CI) *P* value	Model 1 β (95% CI) *P* value	Model 2 β (95% CI) *P* value	Model 3 β (95% CI) *P* value
Per 1 ug/dL increase	0.046 (-0.053, -0.038) <0.001	-0.016 (-0.021, -0.012) <0.001	-0.011 (-0.015, -0.007) <0.001	-0.026 (-0.033, -0.019) <0.001	-0.015 (-0.020, -0.010) <0.001	-0.008 (-0.012, -0.003) 0.001	-0.022 (-0.028, -0.016) <0.001	-0.014 (-0.018, -0.009) <0.001	-0.006 (-0.010, -0.002) 0.005
BLL (Quartile)									
Q1 (0.18-0.59)	Reference	Reference	Reference	Reference	Reference	Reference	Reference	Reference	Reference
Q2 (0.59-0.82)	-0.036 (-0.050, -0.022) <0.001	-0.008 (-0.017, 0.000) 0.052	-0.004 (-0.012, 0.003) 0.266	-0.007 (-0.020, 0.006) 0.283	-0.002 (-0.011, 0.007) 0.592	0.003 (-0.010, 0.008) 0.812	-0.006 (-0.018, 0.006) 0.304	-0.002 (-0.011, 0.006) 0.597	0.003 (-0.005, 0.011) 0.437
Q3 (0.82-1.20)	-0.071 (-0.085, -0.057) <0.001	-0.024 (-0.032, -0.015) <0.001	-0.014 (-0.022, -0.006) <0.001	-0.034 (-0.047, -0.020) <0.001	-0.021 (-0.030, -0.012) 0.001	-0.007 (-0.026, 0.008) 0.114	-0.033 (-0.045, -0.020) <0.001	-0.022 (-0.031, -0.013) <0.001	-0.008 (-0.016, -0.001) 0.048
Q4 (>1.20)	-0.106 (-0.121, -0.091) <0.001	-0.031 (-0.040, -0.021) <0.001	-0.018 (-0.027, -0.010) <0.001	-0.058 (-0.072, -0.044) <0.001	-0.031 (-0.041, -0.021) <0.001	-0.013 (-0.022, -0.004) <0.001	-0.050 (-0.063, -0.037) <0.001	-0.028 (-0.037, -0.018) <0.001	-0.010 (-0.018, -0.001) 0.029
*P* for trend	<0.001	<0.001	<0.001	<0.001	<0.001	0.002	<0.001	<0.001	0.005
Stratified by age									
8–13 years	-0.025 (-0.032, -0.018) <0.001	-0.025 (-0.032, -0.018) <0.001	-0.014 (-0.020, -0.008) <0.001	-0.014 (-0.021, -0.008) <0.001	-0.023 (-0.029, -0.016) <0.001	-0.010 (-0.015, -0.004) <0.001	-0.011 (-0.017, -0.005) <0.001	-0.020 (-0.026, -0.014) <0.001	-0.007 (-0.012, -0.002) <0.001
14-19 years	-0.017 (-0.025, -0.009) <0.001	-0.013 (-0.021, -0.005) 0.001	-0.013 (-0.020, -0.006) <0.001	0.005 (-0.004, 0.014) 0.320	-0.015 (-0.023, -0.006) 0.001	-0.011 (-0.019, -0.004) 0.004	0.003 (-0.006, 0.012) 0.539	-0.012 (-0.021, -0.003) 0.006	-0.009 (-0.017, -0.002) 0.015
Test for interaction	0.158	0.025	0.833	<0.001	0.142	0.705	<0.001	0.142	0.579
Stratified by sex									
Men	-0.021 (-0.031, -0.012) <0.001	-0.011 (-0.016, -0.005) <0.001	-0.008 (-0.012, -0.003) <0.001	-0.020 (-0.029, -0.011) <0.001	-0.013 (-0.019, -0.007) <0.001	-0.008 (-0.014, -0.002) 0.006	-0.017 (-0.025, -0.009) <0.001	-0.012 (-0.018, -0.006) <0.001	-0.007 (-0.012, -0.002) 0.002
Women	-0.079 (-0.091, -0.067) <0.001	-0.029 (-0.037, -0.021) <0.001	-0.019 (-0.026, -0.012) <0.001	-0.060 (-0.070, -0.049) <0.001	-0.024 (-0.032, -0.016) <0.001	-0.011 (-0.018, -0.004) <0.001	-0.050 (-0.060, -0.040) <0.001	-0.020 (-0.028, -0.012) <0.001	-0.007 (-0.014, 0.001) 0.054
Test for interaction	<0.001	<0.001	0.006	<0.001	<0.030	0.523	<0.001	0.117	0.974
Stratified by race/ethnicity									
Mexican American	-0.028 (-0.038, -0.017) <0.001	0.019 (-0.026, -0.013) <0.001	-0.013 (-0.019, -0.007) <0.001	-0.014 (-0.024, -0.004) 0.005	-0.015 (-0.022, -0.008) <0.001	-0.007 (-0.013, -0.001) 0.017	-0.014 (-0.023, -0.005) 0.001	-0.015 (-0.022, -0.009) <0.001	-0.007 (-0.013, -0.002) 0.012
Other Hispanic	-0.035 (-0.059, -0.012) 0.003	-0.012 (-0.026, 0.002) 0.103	-0.011 (-0.024, 0.002) 0.103	-0.016 (-0.037, 0.005) 0.129	-0.011 (-0.026, 0.003) 0.134	-0.011 (-0.024, 0.003) 0.136	-0.007 (-0.026, 0.012) 0.473	-0.002 (-0.017, 0.013) 0.792	-0.002 (-0.015, 0.012) 0.823
Non-Hispanic White	-0.051 (-0.067, -0.034) <0.001	-0.015 (-0.025, -0.005) 0.003	-0.011 (-0.020, -0.001) 0.024	-0.031 (-0.046, -0.015) <0.001	-0.018 (-0.029, -0.007) <0.001	-0.010 (-0.020, -0.001) 0.042	-0.027 (-0.041, -0.014) <0.001	-0.017 (-0.027, -0.006) 0.001	-0.009 (-0.019, -0.001) 0.048
Non-Hispanic Black	-0.068 (-0.080, -0.057) <0.001	-0.019 (-0.026, -0.011) <0.001	-0.012 (-0.019, -0.005) <0.001	-0.047 (-0.057, -0.036) <0.001	-0.013 (-0.021, -0.005) 0.001	-0.005 (-0.012, 0.002) 0.165	-0.042 (-0.051, -0.032) <0.001	-0.013 (-0.020, -0.005) 0.001	-0.005 (-0.012, -0.002) 0.183
Other Race Test for interaction	-0.046 (-0.085, -0.007) 0.022 <0.001	-0.002 (-0.025, 0.021) 0.880 0.556	0.004 (-0.018, 0.025) 0.733 0.637	-0.024 (-0.060, 0.012) 0.186 0.027	-0.004 (-0.029, 0.021) 0.755 0.748	-0.001 (-0.022, 0.023) 0.976 0.834	-0.013 (-0.045, 0.020) 0.441 0.017	0.006 (-0.018, 0.029) 0.646 0.231	0.012 (-0.009, 0.032) 0.272 0.334

Model 1 unadjusted. Model 2 adjusted for age, gender, and race/ethnicity. Model3 adjusted for age, gender, and race/ethnicity, PIR, body mass index, serum calcium, serum phosphorus. BLL Blood lead levels. BMD bone mineral density. PIR poverty income ratio.

**Table 3:** Results of weighted linear regression modeling for associations of the BLL with BMD at different sites after excluding individual blood lead more than 3.5 ug/dl.

	Model 1 β (95% CI) *P* value	Model 2 β (95% CI) *P* value	Model 3 β (95% CI) *P* value
Total spine	-0.069 (-0.080, -0.059) <0.001	-0.022 (-0.029, -0.016) <0.001	-0.014 (-0.020, -0.008) <0.001
Total femur	0.039 (-0.049, -0.030) <0.001	-0.024 (-0.031, -0.017) <0.001	-0.012 (-0.018, -0.005) <0.001
Femur neck	-0.033 (-0.042, -0.024) <0.001	-0.021 (-0.027, -0.014) <0.001	-0.009 (-0.015, -0.003) 0.004

Model 1 unadjusted. Model 2 adjusted for age, gender, and race/ethnicity. Model3 adjusted for age, gender, and race/ethnicity, PIR, body mass index, serum calcium, serum phosphorus. BLL Blood lead levels. BMD bone mineral density. PIR poverty income ratio.

#### Stratified Analyses by Age, Gender, and Race/Ethnicity

Stratified analyses were performed by age (8-13 and 14-19) (**Table 2**). In the fully adjusted models, the negative correlation was also significant in ages between 8-13 and 14-19 years at all sites of interest, with no interactive effect. In aged between 8-13, the BLL was negatively correlated to total spine BMD (β= -0.014 95% CI: -0.020, -0.008, *P*<0.001), total femur BMD (β= -0.010 95% CI: -0.015, -0.004, *P*<0.001), and femur neck (β= -0.007 95% CI: -0.012, -0.002, *P*<0.001). In aged between 14-19, the BLL was negatively correlated to total spine BMD (β= -0.013 95% CI: -0.020, -0.006, *P*<0.001), total femur BMD (β= -0.011 95% CI: -0.019, -0.004, *P*=0.004), and femur neck (β= -0.009 95% CI: -0.017, -0.002, *P*=0.015). For gender, the negative correlation was also significant in males and females at all sites of interest in the fully adjusted models. Effect estimates were stronger in girls than boys at the lumbar spine (*P* for interaction= 0.006). For race/ethnicity, the negative association between BLL and BMD was pronounced in Mexican American, Non-Hispanic White, and Non-Hispanic Black in the fully adjusted models, but not in Other Hispanic and Other Races (Including Multi-Racial). However, no interactive effects were observed.

## Discussion

The present study found a negative association between BLL and BMD at the spine and femur in children and adolescents. For every 1mg/dl increase in BLL, the BMD of the total spine, total hip, and femoral neck decreased by 0.011 g/cm^2^, 0.008 g/cm^2^, and 0.006 g/cm^2^, respectively. The results showed that Pb affected the lumbar spine more than the femur. The effect estimates were stronger in girls than boys at the lumbar spine. This association remained significant in American children and adolescents after excluding participants with BLL more than 3.5 ug/dl. It means that even at the reference concentration (<3.5 ug/dl), BLL still has a negative correlation with BMD in children and adolescents.

Pb has been proved to be a hazardous metal toxic to many organs and systems, including the kidneys, bones, blood system, digestive system, nervous system, and so on (26). BLL in Americans has been reported to decline in recent years (27). In 2012, the Centers for Disease Control (CDC) identified blood lead reference value (BLRV) as 5.0 ug/dl using the BLL distribution in American children aged 1–5 years from NHANES 2007-2010. Then, CDC updated BLRV in children to 3.5 µg/dL using NHANES 2015-2018 (25). The mean concentration of BLL in our study was 1.04 ± 0.87 ug/dl, which was lower than in previous studies (17, 28). Among the 5,583 subjects, only 103 (1.84%) participants were above 3.5μg/dL. Nevertheless, our study demonstrated that Pb exposure had an adverse effect on BMD in children and adolescents, even at a low level (<3.5 µg/dL). Cumulative evidence has suggested that there was no safe BLL for children since even very low levels cause harm (29). Further actions are needed to be taken by individuals, healthcare providers, and policymakers to eliminate Pb exposure among children and adolescents, especially in some areas with potential threats (30).

Many studies have explored the molecular mechanism between Pb and bone metabolism. *In vitro*, experiments showed that Pb could inhibit the normal physiological metabolism of chondrocytes and osteoblasts (31, 32). In addition, Pb could inhibit the function of active vitamin D and the absorption of calcium from the diet (33). Moreover, Pb could interfere with normal bone metabolism through competitive inhibition of osteocalcin (34). Studies investigating the association of Pb exposure and BMD have drawn different conclusions for adults (10, 15, 35). Lu et al. (15) found that BLL was negatively associated with BMD in American female adults. However, this negative correlation was not found in men. Another study using the NHANES database showed a negative association of blood lead and urine lead concentrations with BMD in American women older than 40 years (10). Wei et al. (35) found that Pb exposure was negatively associated with BMD in American adults aged ≥20 years. Studies that reported the relationship between BLL and BMD in children and adolescents were scarce and inconsistent. An early study measuring BMD using DXA at one-third of the radius, investigated the association between BLL and BMD in American children (28). They included 59 black children who attended the Medical and Lead Poisoning Clinic. However, this small-sample study did not find any association between BLL and BMD. In another early cross-sectional study by James et al. (17), they recruited African American children aged 8-10 years for analysis. The children were divided into two cohorts by BLL. They found that the cohort with high BLL had higher BMD, which was contrary to the finding of our study. They made some explanations for the results. Pb can inhibit the parathyroid hormone-related peptide (PTHrP) and transform growth factor-β1, leading to the chondrocyte precocity (36). However, the higher BMD associated with the PTHrP suppression was transient. Although PTHrP-deficient mice were born with higher BMD, their BMD got lower in later years (37). Different study populations, designs, and statistical methods could explain these inconsistent results. First, the two studies included a small sample size, which can affect the reliability of the results. Moreover, they only included African American children, which was far from representative because the United States was a multiracial country. Furthermore, these were comparative studies without adjusting relevant variables and performing regression analyses. A recent study conducted by Li et al. (18) found an N-shaped curve relationship between BLL and BMD in children and adolescents. However, there are also some limitations to this study. The outcome variables were lumbar spine BMD, limb BMD, subtotal BMD, and total BMD, but not femur BMD. As we all know, the lumbar spine and femur are usually the sites of most concern to individuals and clinicians regarding osteoporosis (38, 39). The strength of our study was that we included all cycles of NHANES (2005-2010) that included both lumbar spine and femoral bone density data of children and adolescents. In addition, the study did not perform multiple regression analysis, and we could not quantify the relationship between BLL and BMD. Furthermore, we noticed that the study adjusted height and weight, variables with potential multicollinearity in children and adolescents when multivariable generalized additive models (GAMS) were performed. It may lead to inaccurate results.

A study by Campbell JR et al. using data of people older than 50 years from the NHANES database confirmed that Pb exposure was significantly negatively associated with BMD in white subjects, but not in Blacks (16) after adjusting numerous variables. However, we could not determine whether the effect of Pb on BMD was race-specific because no interaction effect was found. Although our results showed that the effects were pronounced in Mexican American, Non-Hispanic White, and Non-Hispanic Black, the effects were not statistically significant for Other Hispanic and Other Races. Further studies with stronger evidence are needed to elucidate the effect of race on Pb exposure and bone mineral density in children and adolescents.

Another remarkable result was that females were more sensitive to Pb exposure than males at the lumbar spine, as proven by the interaction test (*P*= 0.006 for interaction). Several previous studies have come up with the same conclusion for adults (10, 15). Since adolescent women seldom experience menopause, pregnancy, lactation, and so on, further research is needed to explore the underlying mechanisms. Our study also showed that BLL had more effect on the lumbar spine than the femur in American children and adolescents, which was consistent with previous research (40). One explanation is that the trabecular bone (spine) has a larger surface area than cortical bone (femur), causing the spine to absorb more Pb than the femur.

To our knowledge, this is the first large-sample cross-sectional study that found an adverse association between Pb exposure and BMD in children and adolescents. The sample of our study adopts multi-layer random sampling with high reliability and standardization of data, which represents the general population of the United States. However, some limitations should be acknowledged. First, since this is a cross-sectional study, no causal relationship can be inferred. Second, we did not adjust variables such as calcium intake, diet, and exercise, which could bias the results. Third, since DXA is only available to children over 8 years, we were not able to investigate the association between BLL and BMD in children younger than 8 years old.

## Conclusions

Our study indicates that BLL is negatively correlated with BMD at different sites of interest in children and adolescents aged 8-19 years, even in the reference range. In addition, the results show that Pb affects the lumbar spine more than the femur. The effect estimates are stronger in girls than boys at the lumbar spine. Considering the possible adverse effects of Pb exposure on BMD in children and adolescents, individuals, healthcare providers, and policymakers should make efforts to eliminate Pb exposure among children and adolescents. More research is needed to elucidate the relationships between Pb and bone health in children and adolescents, including specific mechanisms and confounding factors like race/ethnicity, gender, and age.

## Data Availability Statement

The raw data supporting the conclusions of this article will be made available by the authors, without undue reservation.

## Ethics Statement

The studies involving human participants were reviewed and approved by the ethics review board of the National Center for Health Statistics. Written informed consent to participate in this study was provided by the participants’ legal guardian/next of kin.

## Author Contributions

Conceptualization, AC, PX, FZ and YZ; Data curation, AC, PX, and BH; Formal analysis, AC, PX, BH, YZM, ZF, HW, FZ, and YZ; Investigation, AC, PX, BH, YZM, ZF, HW, FZ, and YZ; Methodology, AC, PX, and BH; Project administration, AC, PX, FZ and YZ; Software, AC, PX, and BH; Visualization, AC, PX, BH, YZM, ZF, HW, FZ, and YZ; Writing – review & editing, AC, PX, BH, YZM, ZF, HW, FZ, and YZ. All authors contributed to the article and approved the submitted version.

## Conflict of Interest

The authors declare that the research was conducted in the absence of any commercial or financial relationships that could be construed as a potential conflict of interest.

## Publisher’s Note

All claims expressed in this article are solely those of the authors and do not necessarily represent those of their affiliated organizations, or those of the publisher, the editors and the reviewers. Any product that may be evaluated in this article, or claim that may be made by its manufacturer, is not guaranteed or endorsed by the publisher.

## Appendix 1

The associations between BLL and total femur BMD. **(A)** Each black point represents a sample. **(B)** Red line represents the smooth curve fit between variables. Blue lines represent the 95% of confidence interval from the fit. Age, gender, race/ethnicity, PIR, BMI, serum calcium, serum phosphorus were adjusted. Abbreviations: BLL, blood lead levels. PIR, poverty income ratio. BMI, body mass index. BMD, bone mineral density.

## Appendix 2

The associations between BLL and femur neck BMD. **(A)** Each black point represents a sample. **(B)** Red line represents the smooth curve fit between variables. Blue lines represent the 95% of confidence interval from the fit. Age, gender, race/ethnicity, PIR, BMI, serum calcium, serum phosphorus were adjusted. Abbreviations: BLL, blood lead levels. PIR, poverty income ratio. BMI, body mass index. BMD, bone mineral density.
