# Supplementary figures and images for: Blood Lead Level Is Negatively Associated With Bone Mineral Density in U.S. Children and Adolescents Aged 8-19 Years
Source: Front Endocrinol (Lausanne). 2022 Jul 1;13:928752. doi: 10.3389/fendo.2022.928752 (PMC9283721; doi:10.3389/fendo.2022.928752)

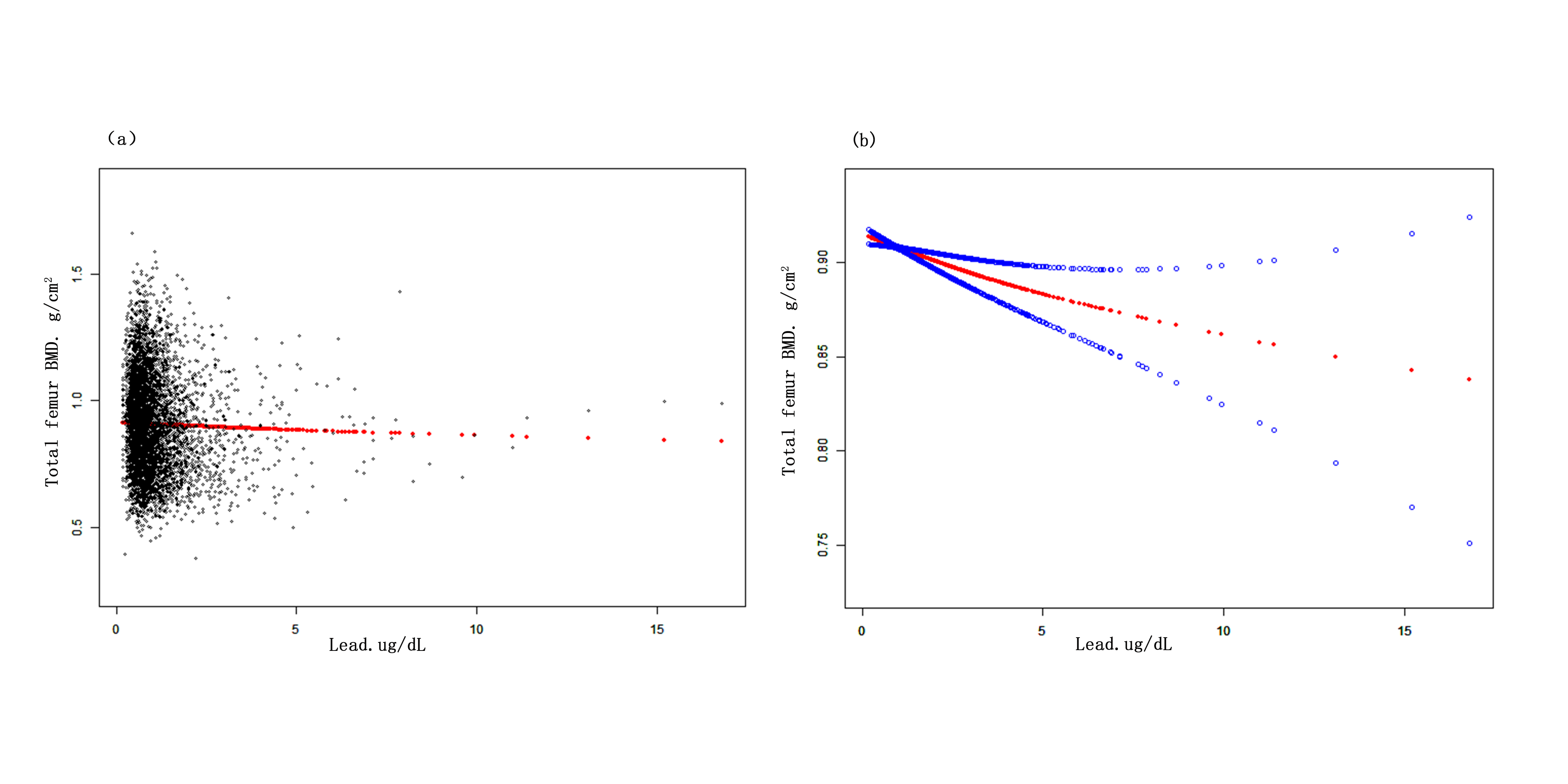

Supplement: Supplementary file 1 [file Image_1.tif]

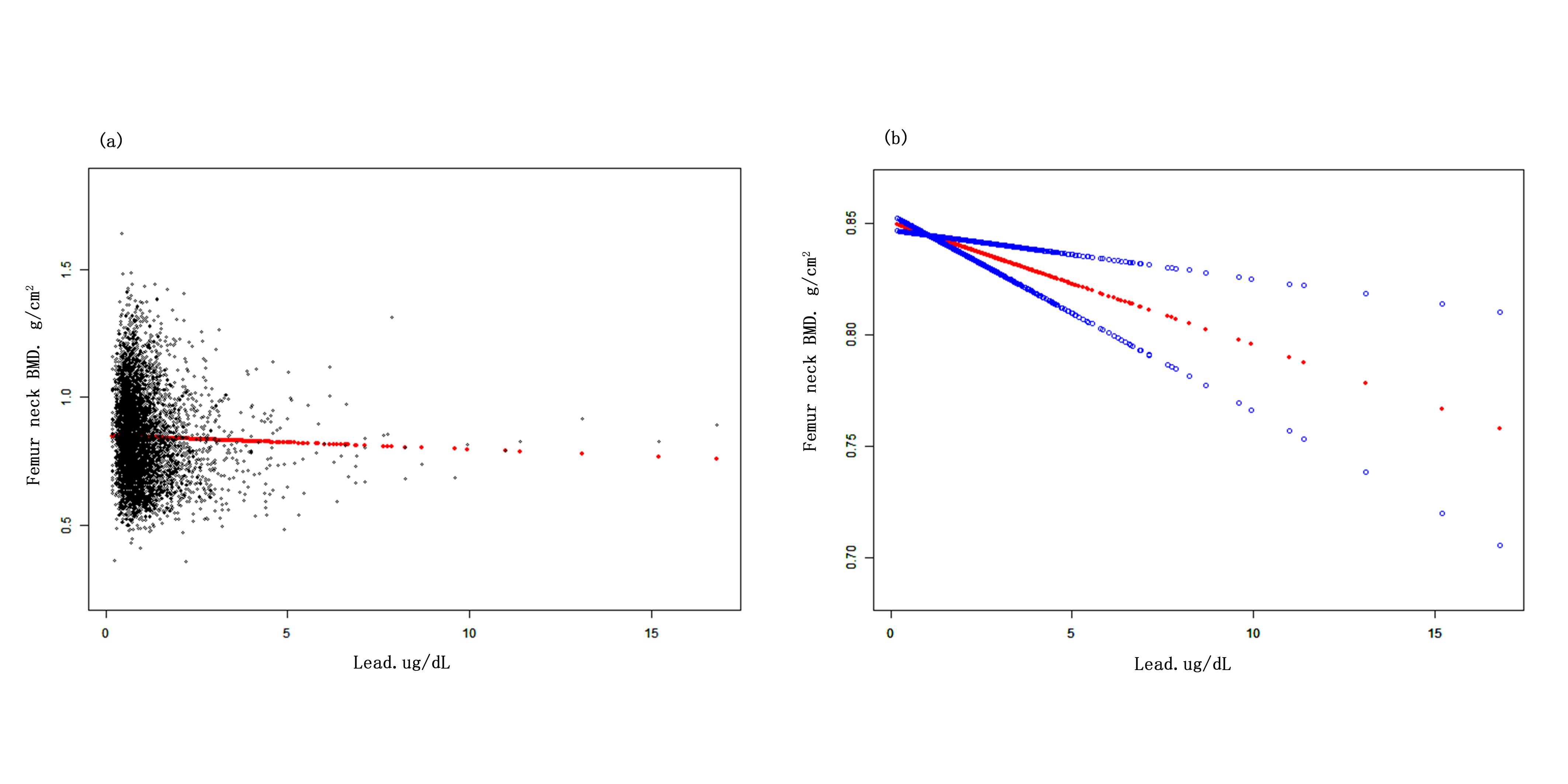

Supplement: Supplementary file 2 [file Image_2.tif]
